# Dynamical facilitation of the ideal free distribution in nonideal populations

**DOI:** 10.1002/ece3.3811

**Published:** 2018-01-31

**Authors:** Garrett M. Street, Igor V. Erovenko, Jonathan T. Rowell

**Affiliations:** ^1^ Department of Wildlife, Fisheries, and Aquaculture Mississippi State University Mississippi State MS USA; ^2^ Department of Mathematics and Statistics University of North Carolina at Greensboro Greensboro NC USA

**Keywords:** cognition, dispersal, facilitation, fitness, modeling, movement, perception, reproduction, simulation

## Abstract

The ideal free distribution (IFD) requires that individuals can accurately perceive density‐dependent habitat quality, while failure to discern quality differences below a given perception threshold results in distributions approaching spatial uniformity. Here, we investigate the role of population growth in restoring a nonideal population to the IFD. We place a simple model of discrete patch choice under limits to the resolution by which patch quality is perceived and include population growth driven by that underlying quality. Our model follows the population's distribution through both breeding and dispersal seasons when perception limits differ in their likely influence. We demonstrate that populations of perception limited movers can approximate an IFD provided sufficient population growth; however, the emergent IFD would be temporally inconstant and correspond to reproductive events. The time to emergence of the IFD during breeding is shorter under exponential growth than under logistic growth. The IFD during early colonization of a community persists longer when more patches are available to individuals. As the population matures and dispersal becomes increasingly random, there is an oscillation in the observance of IFD, with peaks most closely approximating the IFD occurring immediately after reproductive events, and higher reproductive rates producing distributions closer to the IFD.

## 1 INTRODUCTION

The distribution of organisms is determined by the spatial allocation of limiting resources in a given landscape and any competitive pressures from other organisms (Case, Holt, McPeek, & Keitt, 2005; Holt & Keitt, 2005; Lima & Zollner, 1996; McLoughlin, Morris, Fortin, Vander Wal, & Contasti, 2010). This premise has been classically grounded in the concept of the ideal free distribution (IFD), an equilibrium distribution whereby competitors achieve equal fitness (Fretwell & Lucas, 1969). The IFD arises when (1) individuals accurately observe global variations in the environment, (2) can travel freely, and (3) share equally in local resources. At equilibrium, high‐quality patches will exhibit higher densities of competitors than low‐quality patches such that all individuals are equally fit in their use of resources.

Much effort has been given to testing the IFD in simulation (Farnsworth & Beecham, 1999) and field conditions (Flaxman & DeRoos, 2007; Haugen et al., 2006; Morris, 1997; Oksanen, Power, & Oksanen, 1995; Quaintenne, van Gils, Bocher, Dekinga, & Piersma, 2011), with results generally supporting IFD theory. Analytical studies combining population dynamics with various dispersal rules for improving fitness also support the evolutionary stability of the IFD (Cressman & Křivan, 2006; Cressman, Křivan, & Garay, 2004; Křivan, Cressman, & Schneider, 2008). However, research demonstrating deviation from the IFD has identified cognitive limitations, competitive interactions, and traveling costs as conditions under which the IFD cannot be achieved (Abrahams, 1986; Kennedy & Gray, 1993; Matsumura, Arlinghaus, & Dieckmann, 2010). In particular, Abrahams (1986) demonstrated that, as resource availability declined below a minimum perception threshold (i.e., the minimum resource density at which individuals can detect differences between locations), animals deviate from the IFD and approach uniformity in space use. While competition‐driven deviations from the IFD have been considered (e.g., Cressman et al., 2004; Fretwell & Lucas, 1969; McLoughlin et al., 2010; Reding, Kelley, Rowell, & Rychtář, 2016; Rowell, 2010), perception limits have received little attention beyond Abrahams’ (1986) initial exploration, despite evidence that these perception limits do occur in nature for both food resources (Hakoyama & Iguchi, 1997; Tyler & Clapp, 1995), conspecific presence (Harper, 1982), and predation risk (Polo‐Cavia, Gonzalo, López, & Martin, 2010). Research and management efforts using models and advancements grounded in IFD theory (e.g., habitat selection, species distribution modeling, invasion biology) have thus implicitly, but by necessity, assumed animals can differentiate between high‐ and low‐quality habitat and between those that are under‐ or overutilized.

This raises an interesting question: Can animals be ideally distributed with respect to a resource gradient they cannot perceive? Consider, for example, a species that has colonized a new landscape. Such an organism may be unable to detect differences in resource or habitat quality that its native competitors have evolved to detect, and its population would potentially distribute itself uniformly in accordance with Abrahams (1986), or ‘ideally’ with respect to some perceived quality gradient that does not correspond to reality. Although our hypothetical species would not be distributed ideally, by virtue of occupying habitats that vary in actual quality members of the species could accrue unperceived benefits that would influence survivorship and fecundity. For example, it has been demonstrated that anuran tadpoles do not recognize or respond to chemosensory cues from invasive predators (Polo‐Cavia et al., 2010). Given variable densities of the invasive predator, tadpoles that happen to co‐occur with low predator densities would exhibit higher survivorship than those under high predator densities. Conversely, the distribution of baboons (*Papio cynocephalus ursinus*) is best predicted by the interaction between resource availability and perceived predation risk, invoking a trade‐off between risk management and foraging efficiency (Cowlishaw, 1997), but baboons that select against perceived risk in low predator density areas could thus incur an unnecessary fitness loss due to reduced foraging success. In either case, each population might exhibit an IFD driven by the true gradient of predation risk despite having selected only for perceived predation risk; that is, an IFD may emerge as those animals that have by chance found themselves in higher quality habitat experience higher fecundity and survivorship than their less fortunate competitors.

The implication of such a scenario is that population dynamics alone may give rise to the IFD in the absence of ideally motivated searchers. This has been analytically supported in populations with immobile or low mobility organisms (Cressman & Křivan, 2006; see also Hastings, 1983; Levin, Cohen, & Hastings, 1984). Movement by highly mobile organisms could overcome the contribution of population dynamics to establishing an IFD, particularly if they imperfectly perceive habitat quality. One may thus expect that, given a discrete set of patch choices of variable density‐dependent quality and a minimum quality perception threshold, an ideal searcher would begin to exhibit nonideal behavior at higher competitor densities (i.e., overutilized habitat), consistent with existing theory (Abrahams, 1986; Kennedy & Gray, 1993; Matsumura et al., 2010). Alternatively, deviation from the IFD due to poor perception may be mitigated by increased true habitat quality increasing reproductive success, but it is not clear how persistent such facilitation would be over time. Extended periods of movement between reproductive events for nonideally behaving organisms should allow the population to more closely resemble uniformity in space use (Tyler & Hargrove, 1997); thus, any benefit of population growth to achieving the IFD may diminish with time since reproduction. Further, the rate of population growth should also influence the rate of emergence of the IFD because higher intrinsic growth rates, or higher local growth rates driven by increased habitat quality, will produce a greater number of animals in high‐quality habitat than in low quality. This implies a balance between the rate and extent of organismal movement and the rate of population growth in determining whether dynamical facilitation of the IFD can occur.

Our objective here was to investigate the role of population dynamics in restoring a nonideal population to the IFD. We constructed an agent‐based stochastic simulation of discrete patch choice under a perception limit following the example in Abrahams (1986). We also simulated population dynamics under both exponential (density independent) and logistic (density dependent) growth scenarios. We evaluated the emergent seasonal distributions of organisms under both growth scenarios over time and compared their similarity to the expected IFD. Operating under the hypothesis that population growth serves to drive populations toward the IFD, we predicted that (1) populations of nonideal movers would achieve an IFD provided sufficient population growth; (2) the emergent IFD would be temporally inconstant and correspond to reproductive events; (3) time to emergence of the IFD would be shorter under exponential growth than under logistic growth; and (4) that initial maintenance of an IFD is prolonged during dispersal as the number of possible sites increases.

## 2 MATERIALS AND METHODS

We simulated the evolving distribution of a population of individual animals over an array of *k* discrete patches, *P*_i_, via an agent‐based model. In this model, each patch provides a continuously renewable, constant standing resource, *R*_i_, and hosts a local population, *n*_i_. All residents perform identically and equitably share in the patch's resources. The corresponding suitability of the patch is the per capita fitness, *R*_i_/*n*_i_.

This stochastic simulation extends the framework previously proposed by Abrahams (1986). It incorporates not only dispersal under limited perception but also reproductive events. Beginning with an initial colonization of the environment by *N*_0_ individuals, the model alternates between seasons of dispersal and reproduction (detailed below). Seasonal lengths are defined by the number of events that occur, which is a function of the total population level $N=\sum_{i=1}^{k}n_{i}$. Every simulation run completes five full dispersal‐breeding cycles before terminating. (The first dispersal season corresponds to the initial colonization of the environment.) Additionally, we also considered the behavior of the population with dispersal and reproduction co‐occurring.

### 2.1 Dispersal

During the first dispersal season, the environment is initially empty, and *N*_0_ individuals sequentially colonize the landscape from an external source location. In all subsequent seasons, the number of dispersal events equal the current size of the total population *N*. For each dispersal event, an individual is selected at random with equal probability so that each animal, on average, has the opportunity to move once per season. This individual vacates its current site *P*_c_ ($n_{c}\to n_{c}-1$) and then evaluates the state of the environment.

All animals share a quantifiable perception limit, PL. Two patches, *P*_i_ and *P*_j_, are perceived to be of equal suitability when the difference between their potential suitabilities drops below this threshold, that is,

(1) $$\left|\frac{R_{i}}{n_{i}+1}-\frac{R_{j}}{n_{j}+1}\right|\leq \text{PL}.$$

If the most suitable patch, *P*_M_, is perceptually distinct from all other sites, then the individual settles there (*n*_M_ → *n*_M_ + 1). Otherwise, it randomly selects with uniform probability a patch *P*_j_ from the set of patches indistinguishable from *P*_M_ (*n*_j_ → *n*_j_ + 1). As populations increase over time, the differences between patches grow small and consistently fall below the perception limit.

### 2.2 Reproduction

The number of events in each reproductive season is given as a function of the population size at the start of the breeding season, *f*(*N*). The reproducing individual is drawn randomly from the population, weighted by the individual's fitness, *R*_i_/*n*_i_. The resulting offspring is then placed at the site of the parent (*n*_i_ → *n*_i_ + 1).

We ran simulations for two different interpretations of the growth function *f*(*N*). In our first series of simulations, we assumed unbounded exponential growth, with *f*(*N*) = *rN*. Here, the parameter *r* is the average reproductive rate (offspring per individual per season). In our second series, the number of reproductive events is set by the logistic function *f*(*N*) = *r N*(1‐*N*/*K*) where *K* is the carrying capacity of the total population. In the logistic growth case, we stopped the simulation once the total population level was within 1% of the carrying capacity *K*. In this scenario, the number of reproductive events (i.e., breeding seasons) will increase with *K* due to increased time required for *N* to saturate at *K*; thus, the number of breeding events is determined by the specific parameterization of the logistic function.

In the simplest 2‐patch scenario (i.e., *k* = 2), we assumed that resources were distributed as a ratio, ranging from 2:1 (*R*_1_ = 12 and *R*_2_ = 6) to 6:1 (*R*_1_ = 18 and *R*_2_ = 3) for the main set of simulations. In the 10‐patch scenario, the resources were distributed as follows: *R*_1_ = 12, *R*_2_ = 11, *R*_3_ = 10, *R*_4_ = 9, *R*_5_ = 8, *R*_6_ = 6, *R*_7_ = 5, *R*_8_ = 4, *R*_9_ = 3, and *R*_10_ = 2, resulting in the 6:1 ratio between the richest and the poorest patch. In both *k* = 2 and *k* = 10 scenarios, colonization was performed by *N*_0_ = 10 individuals, and we ran 1000 trials for each combination of parameters and averaged the results.

## 3 RESULTS

### 3.1 Exponential growth

**Figure 1:**
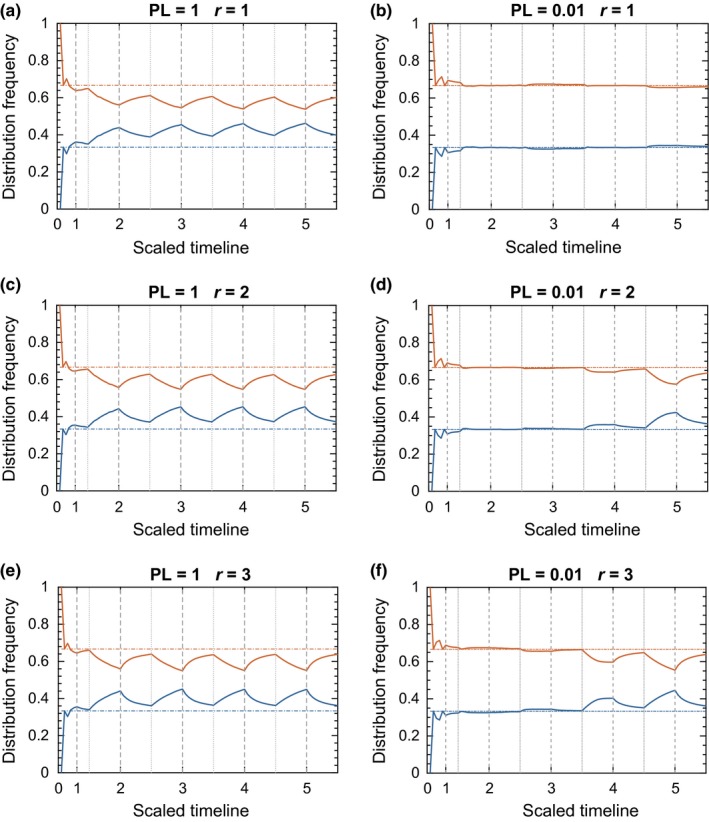
Results of the 2‐patch simulation for the exponential growth model. The relative distribution of the population across the two patches changes over time, with the distribution approaching IFD during periods of breeding and diverging during dispersal once the community has achieved a size sufficient to impose perception limits. The seasonal time scale is marked at the start of each breeding season (1–5). (Note, *T* = 0 marks the initial colonization rather than the onset of reproduction, hence its shorter interval). Dash‐dotted horizontal lines show IFD levels. Solid lines show actual distribution of animals between the two patches. Color code: orange for rich patch, blue for poor patch. Vertical dashed lines indicate starting points of reproductive seasons. Vertical dotted lines indicate starting points of dispersal periods

We simulated the evolution of a population under exponential growth using two levels of the perception limit (PL = 1.0 and 0.01) and three reproductive growth rates (*r* = 1, 2, and 3; Figure 1) in a 2‐patch system. As the population matured, the densities in the patches approximated a periodic, season‐linked behavior that oscillated in its approach toward ideal (breeding) and uniform (dispersal) densities. The perception limit was effectively imposed on a continual basis. The animals subsequently lost their ability to distinguish between optimal and inferior patches and drifted to uniform distribution during dispersal.

As the reproductive rate increased, two phenomena were observed. First, the onset of seasonal periodicity developed sooner. This may not be as obvious when animals have coarse perception (PL = 1.0), but the effect is evident with more discriminating populations (PL = 0.01). Second, populations with greater reproduction more closely approximate the ideal free distribution at the end of the breeding season.

**Figure 2:**
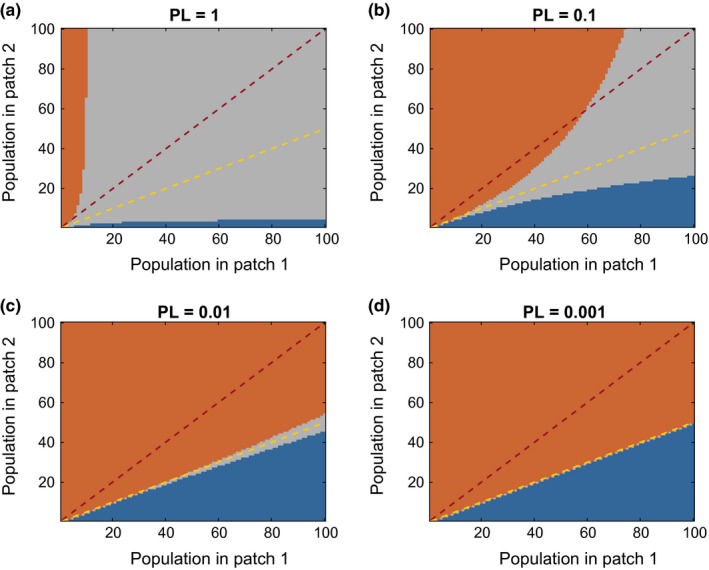
Directed and random movement. The distribution of the population between the two patches affects whether movement is directed toward an ideal distribution or a uniform one. The onset of random movement is delayed until larger population levels are attained with a narrower deviation from the ideal as the perception limit decreases. Color code: orange region—rich patch is preferred, blue region—poor patch is preferred, gray region—individuals cannot distinguish between the quality of the two patches, red dashed line—uniform distribution, gold dashed line—ideal free distribution

In contrast, as the perception limit became smaller, the animals retained their ability to distinguish between sites for a greater length of time, and the population prolonged its near‐ideal distribution for larger population sizes (Figure 2). This effect is best illustrated in a population of highly perceptive individuals with low fecundity, PL = 0.01 and *r* = 1.

Although the selection of a reproducing adult is determined by the individual's share of resources (*R*_i_/*n*_i_), animals residing in the first patch, in aggregate, maintained a 2‐to‐1 advantage over the poor patch.

**Figure 3:**
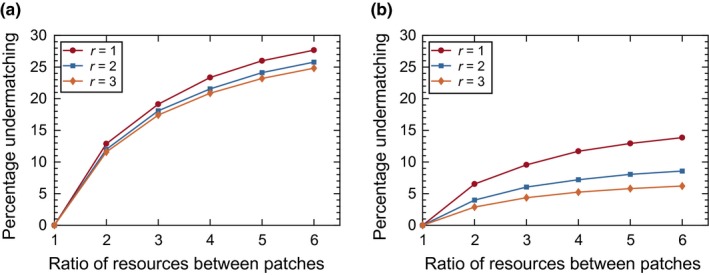
Effect of different resource ratios between two patches on the facilitation of the IFD in the exponential growth model with coarse perception (PL = 1.0). (a) The percentage of the population residing in the poor patch in addition to IFD levels at the end of the last dispersal season. (b) The percentage of the population residing in the poor patch in addition to IFD levels at the end of the last reproduction season

We also considered different ratios of resource allocations between the two patches. For our main simulations, the ratio of resources was 2:1, and we extended this to 3:1, 4:1, 5:1, and 6:1 ratios in the coarse perception (PL = 1.0) case. Unsurprisingly, we found that increasing the resource ratio between patches makes it more difficult to achieve the IFD (Figure 3). The percentage of the population residing in the poor patch in addition to the level predicted by IFD (usually referred to as “undermatching’’) grows as the difference in the quality between the patches increases.

**Figure 4:**
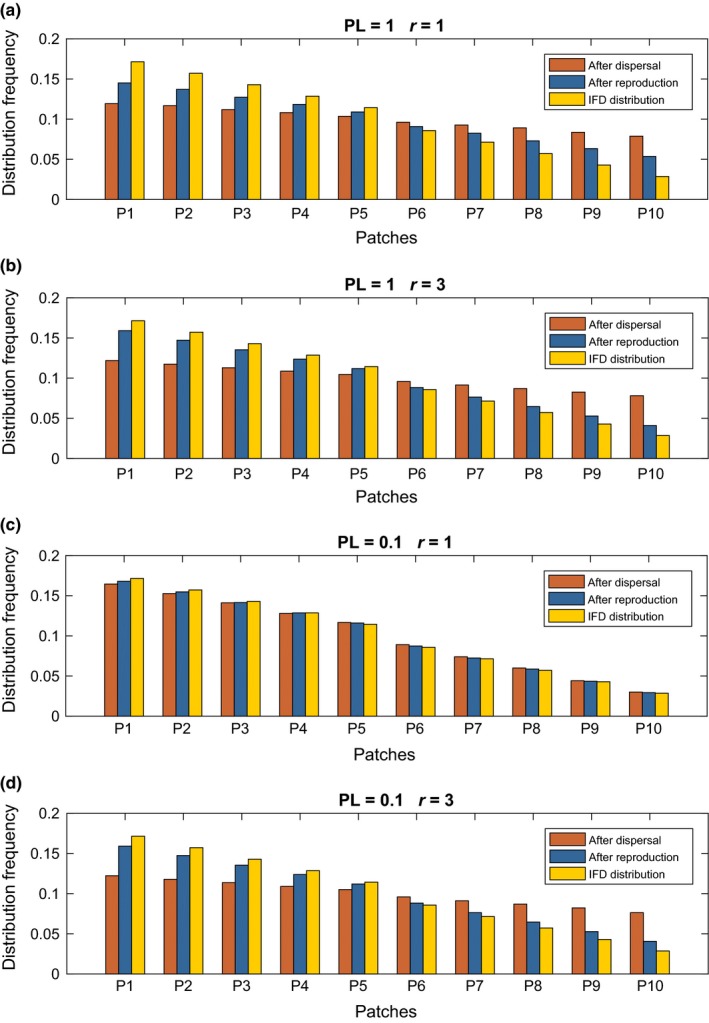
Results of the 10‐patch simulation for the exponential growth model at the end of the last dispersal‐reproduction season (season 5). Resource quality declines from patch P_1_ to P_10_. The average density of individuals in a given patch at the end of the final dispersal phase (orange bar) is always further from the expected IFD density (yellow bar) than is the average density at the end of the final reproduction phase (blue bar)

Next, we repeated our simulations of dispersal and exponential growth but increased the number of patches from 2 to 10. We observed the same qualitative behavior in the 10‐patch simulation as the 2‐patch simulation; that is, lower perception limits resulted in animals prolonging their near‐ideal distribution, but ultimately distributions deviated from IFD most strongly after dispersal and returned to near‐IFD after reproduction (Figures 4, S1–S3). However, deviation from IFD was more difficult to detect due to decreased difference in quality between patches (Figs S1–S3).

We also simulated continuous, rather than seasonal, reproduction. Similar to the discrete reproduction models, $N_{0}=10$ individuals are allowed to initially colonize the environment. After the initial colonization is complete, a series of reproduction and dispersal events occur. The type of the next event (reproduction or dispersal) is chosen at random; the probability that the next event is reproduction is equal to *r*/(*r* + 1), where *r* is the reproductive rate, and the probability that the next event is dispersal is equal to 1/(*r* + 1). The total number of events is set equal to that for the discrete reproduction model with exponential growth of rate *r*. With this setup, the ratio of reproductive to dispersal events in the continuous model is identical to that for the discrete model with exponential growth.

**Figure 5:**
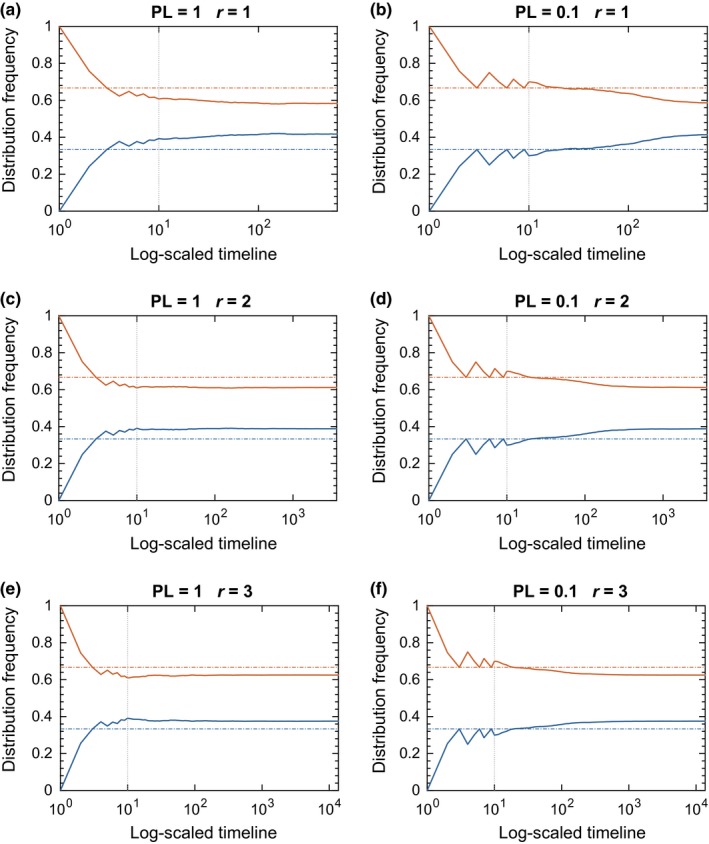
Results of the simulation for the continuous reproduction model. The event timeline is log‐scaled. The numbers on the *x*‐axis indicate the actual number of events in the event timeline. Dash‐dotted horizontal lines show IFD levels. Solid lines show actual distribution of animals between the two patches. Color code: orange for rich patch, blue for poor patch. The vertical dotted line indicates the end of the initial colonization period and the beginning of the dispersal‐reproduction events

The results of the simulation are summarized in Figure 5. We observe that once the population density reaches the level where the animals cannot distinguish between the two patches, the resulting distribution stabilizes at levels identical to those in the seasonal reproduction scenario (cf. Figure 1).

### 3.2 Logistic growth

**Figure 6:**
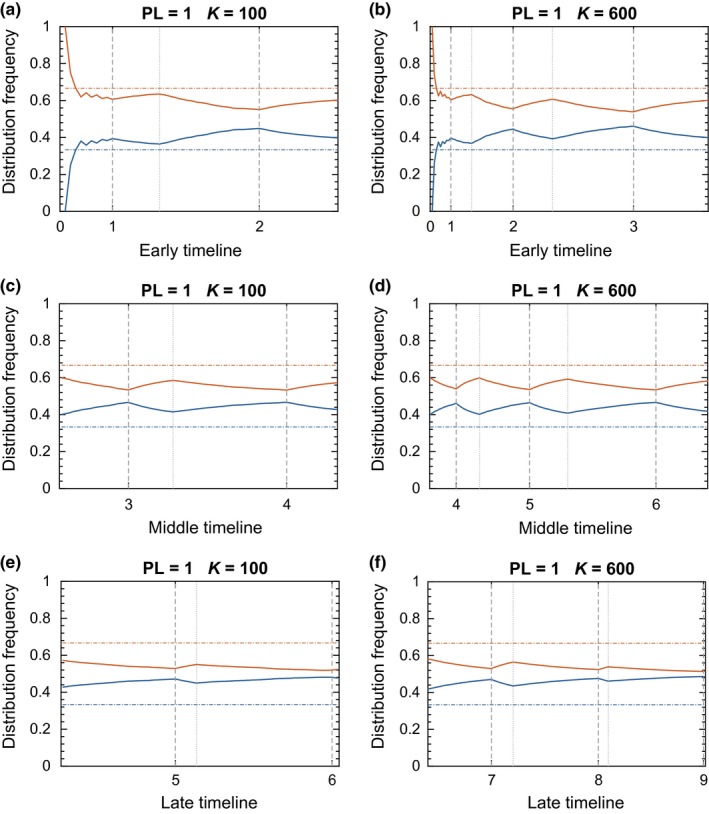
Results of the 2‐patch simulation for the logistic growth model. The entire simulation is broken down into three parts: early, middle, and late. Each part contains an equal number of reproductive seasons. The numbers 1–6 (or 1–9) on the *x*‐axis indicate the starting points of the corresponding reproductive seasons in the event timeline; 0 indicates the starting point of the simulation before the initial colonization of the environment. Dash‐dotted horizontal lines show IFD levels. Solid lines show actual distribution of animals between the two patches. Color code: orange for rich patch, blue for poor patch. Vertical dashed lines indicate starting points of reproductive seasons. Vertical dotted lines indicate starting points of dispersal periods

For our simulations of the logistic growth model, we kept the perception limit constant at the coarse scale value PL = 1.0 and set *r* = 1 for all simulations. We considered two values of the carrying capacity, *K* = 100 and *K* = 600. The former was simulated through six (6) reproductive seasons, while the latter extended to nine (9) seasons due to the increased time required for the population size *N* to saturate at *K*. We partitioned the data from these simulations into three phases, each containing an equal number of reproductive seasons (2 and 3, respectively; Figure 6).

In the early phase of each run, the number of both movement and reproductive events was comparatively small (cf. exponential growth with *r* = 1). The population was relatively ideal through the first cycle and then began the oscillatory approaches to uniform and ideal distributions with the start of the second cycle. The total number of reproductive events was greatest in the middle phase (cycles 3–4 and 4–6, respectively). There was a slight decline in the maximum approximation to the IFD over successive breeding seasons. In the terminal phase of the simulations, reproductive growth plummeted as the population approached carrying capacity. Dispersal events dominated this phase of the population. Once reproduction ceased, the population consistently drove toward a uniform distribution. The carrying capacity played a role in the number of seasons required to reach the dispersal‐only period; however, it did not qualitatively affect the early‐middle‐late patterning in the population's evolution.

### 3.3 Distributions for large populations

For large or nondiscrete populations (e.g., population densities), a similar oscillation can be observed between more uniform distributions at the close of the dispersal period and more ideal distributions at the end of the breeding period. If we assume a rate of *m* movement events per individual during dispersal, the rate of change in patch populations, *u* and *v*, respectively, is given by

(2) $${\left(\begin{array}{c} u\\ v \end{array}\right)}^{\prime }=\left[\begin{array}{cc} -m/2 & m/2\\ m/2 & -m/2 \end{array}\right]\left(\begin{array}{c} u\\ v \end{array}\right)$$

After dispersal, there are *r* reproductive events per individual on average, with patch recruitment proportional to patch quality *P*_i_. Total quality is *P*_T_ = *P*_1_ + *P*_2_. The net effect of these two processes is an annual census update. Letting *u*_n_ and *v*_n_ be the patch populations at the start of dispersal, the community at the start of the next dispersal season is given as

(3) $$\begin{array}{c} u_{n+1}=\left(\frac{P_{T}+2rP_{1}}{2P_{T}}\right)\left(u_{n}+v_{n}\right)+\left(\frac{u_{n}-v_{n}}{2}\right)e^{-m},\\ v_{n+1}=\left(\frac{P_{T}+2rP_{2}}{2P_{T}}\right)\left(u_{n}+v_{n}\right)-\left(\frac{u_{n}-v_{n}}{2}\right)e^{-m}. \end{array}$$

The distribution frequency of the population pre‐ and post‐dispersal stabilizes within five to seven iterations for all parameter combinations (*m* = 1 to 10, *r* = 1–5), which matches our results from the individual‐based model.

## 4 DISCUSSION

Our objective was to investigate the influence of population dynamics in facilitating the appearance of an IFD. Although incorporating population dynamics into the model does not completely overcome the effects of poor cognition, highly fecund animals do exhibit behavior that closely resembles the behavior predicted by the IFD. The cause of this result is that the more valuable patch still has, in toto, proportionately greater output than its alternatives, regardless of the precise individual who produces a given offspring. It does not fully achieve the IFD for two reasons. First, the stochastic nature of the simulation prohibits achievement of the IFD on a consistent, repeatable basis. Second, even in the most fecund of populations, a sizeable portion of the population will be an adult legacy of the preceding dispersal season. For *r* = 3, our most fecund example of exponential growth that still amounts to 25% of the population set in a nonideal arrangement. For similar reasons, dispersal also does not achieve perfect uniformity. As those moving are drawn at random, a nontrivial portion of the total population will not have the opportunity to disperse during the season. This preserves a remnant of the near‐ideal distribution that existed at the start of the season. (This feature is notably absent in the late phase of the logistic growth model.) Yet even provided complete reshuffling of individual positions, those animals in higher quality locations by chance alone would exhibit greater reproductive contributions to net population density, as demonstrated in both seasonal (Figures 1, 6) and continuous breeding models (Figure 5). As such, even under completely random movement, dynamical facilitation of the IFD would still occur (cf. Section 3.3), though possibly to a lesser degree than observed under partial site fidelity as modeled here.

Continual growth is necessary to maintain and restore the near‐IFD condition. Once growth stagnates, as it did with the logistic model, dispersal dominates the behavior of the population (Figure 6). One feature that we did not explicitly model here was mortality; however, it may be that consistent turnover within the composition of the population could mitigate the persistent uniformity in the terminal phase. A common goal in game management is to harvest populations such that maximum offtake is achieved without inducing population decline (i.e., the maximum sustainable yield; MSY). Given density‐dependent population growth, the MSY occurs at the peak of the recruitment curve. Under such a harvest scenario, a population would never achieve carrying capacity, and the IFD could arise based on individual reproductive contributions to population density. This phenomenon might also be expected to occur in species with consistent predation pressure, although variable predation success would be expected to dampen this effect somewhat. This is further complicated by the observation that when between‐patch variation is low, detection of deviations from IFD becomes more difficult (Figs S1–S3). In low‐variation scenarios, it is plausible that one might fail to detect deviations from IFD, particularly without knowing the true ideal distribution (which is common outside of modeling exercises), and thus falsely conclude that the IFD holds. Our results demonstrate that investigation of the IFD in naturally occurring populations should be conducted (1) immediately following reproductive events to better detect the contribution of population dynamics to achieving the IFD; (2) across levels of population density corresponding to early, middle, or late density dependence (Figure 6); (3) across levels of predation pressure to assess the effect of mortality on arising IFDs; and (4) over multiple seasons to quantify the rate of deviation from the IFD due to movement and to characterize time lags in reproductive success. Additionally, we suggest (5) that deviations from and restorations to IFD will be more observable in areas with as few patches as possible while still exhibiting substantial variation in quality.

Although we do not model perception as a heritable trait subject to selection in this current paper, animals that can detect finer gradations in quality should have increased fitness relative to their cohort (Koops & Abrahams, 2003), provided that perception is differentiably expressed. This important caveat narrows the temporal window on which selection is active. We conjecture that selection for improved perception would occur during the intermediate phase of a community's development after colonization has allowed the population to be widely dispersed but before population growth has saturated the ability of even the most perceptive individuals to discern differences in locations. Selection on perception could also arise in newly emerging systems (e.g., pre‐establishment biological invasions) or when rates of environmental change exceed the rate of generation turnover (i.e., the population cannot adapt as quickly as the environment changes); however, it might also favor less perceptive individuals in environments supporting ecological traps and other risks to dispersers. Once the environment is saturated, poor‐perception individuals will have equal fitness to their improved‐perception peers. This would be particularly pronounced in systems with minimal per‐capita resource heterogeneity, and the final distribution of the perception allele would be determined by the central tendency and variance of resource abundance as they influence adaptive topography (Lande, 2007). Further, in a spatially explicit system where observability is limited, one might see distinct “subpopulations’’ (i.e., clusters of patches with similar allele frequencies) emerge based on resource heterogeneity and local rather than global mean resource abundance.

Our present model has treated only one form of limited perception. Myopia and gradient scale sensing are another form of limitation on the awareness of individuals that occurs in both discretized (Armsworth & Roughgarden, 2005; Cressman & Křivan, 2006) and continuous environments (Cosner, 2005; Reding et al., 2016; Rowell, 2009, 2010). This limitation can establish an IFD within a restricted subrange of the landscape while rendering the distribution nonideal across the larger region, for example, due to crossing resource deserts (Rowell, 2009). Additionally, an inability to observe individual processes such as mortality results in a tension between movement and local dynamics, forming a regional source‐sink dynamic (Abrams, Ruokolainen, Shuter, & McCann, 2012). In addition to these areas, we foresee other forms of limited perception that deserve examination such as the ability to identify resources due to shifting vegetative coverage or the assessment of both the quantity and quality of competitors. Specifically, the density of conspecifics over space is a critical component of IFD theory and is known to influence how animals select for resources in space and by extension their distribution (McLoughlin et al., 2010). Our findings here suggest that an inability to perceive local competitors may promote distribution patterns opposite to that of spatial myopia—that is, individuals may not exhibit an IFD at fine scales owing to increased local conspecific densities arising from detection failure, but may exhibit the IFD at broader scales exceeding the scale of perception where population dynamics come into play (i.e., areas of excessively high conspecific densities induce lower reproductive success). For example, mallards (*Anas platyrhynchos*) may select foraging sites based on perceived habitat quality, but then modify that selection based on subsequent detection of dominant conspecifics (Harper, 1982). This of course assumes that detection of conspecifics signals lower net habitat quality; conversely, the house sparrow (*Passer domesticus*) uses a “chirrup’’ call to notify nearby conspecifics of a divisible resource (Elgar, 1986). Failure to detect calls due to perceptual myopia could in this case evoke scale‐specific IFDs akin to spatial myopia because conspecific detection is an indicator of increased habitat quality. As such, both the ability of an individual to detect conspecifics, and the interpretation of such a detection, require increased attention by spatial and behavioral ecologists.

Although animal space use is decidedly dynamic, with individuals often selecting for seemingly suboptimal habitat at fine spatiotemporal scales, an IFD can emerge from nonideal behavior at both fine (Griffen, 2009) and broad scales (Street, Rodgers, Avgar, & Fryxell, 2015; Street, Rodgers, Avgar, Vander Vennen, & Fryxell, 2017). A necessary next step in understanding this process is to examine how individual movement strategies (e.g., correlated random walks vs. Brownian motion; Turchin, 2015), and variation among individuals in movement behaviors (e.g., exploratory vs. site‐fidelitous), encourage or inhibit the development of an IFD in a spatially explicit population with variable perception. A generally nonideal population with limited perception should approach spatial uniformity as described here, but the rate of return toward an IFD following reproduction would be dependent on the spatial arrangement of resources interacting with local population densities (McLoughlin et al., 2010) and the rate of dispersal through the landscape (Turchin, 2015). This would likely be mitigated by memory permitting the animal to return to previously identified high‐quality locations (Avgar et al., 2015), suggesting that spatial memory may be capable of overcoming the influence of perception thresholds on the IFD to some degree. Further investigation of these interrelated effects—movement, spatial memory, perception, and population dynamics—is needed to determine the relative contribution of each to realized animal distributions in a spatially explicit system.

Our finding that dispersal is a source of deviation from the IFD echos earlier works. Diffusion across spatially varying landscapes drives populations further away from the IFD as movement increases, with sedentary populations preserving the ideal community structure (Hastings, 1983; Levin et al., 1984). Other contributions to the literature have examined how the IFD emerges or fails given other complicating factors, including competitive pressures (Lou, Tao, & Winkler, 2014), the inability to detect global habitat quality (Cressman & Křivan, 2006), or when movement is maladaptive (Abrahams, 1986; Galanthay & Flaxman, 2012). Our study bridges the extremes of dispersive behavior by offering a density‐dependent mechanism—the perception limit—by which a population first strives toward and then subsequently diverges from an ideal distribution, with a secondary process reestablishing the IFD through seasonal breeding.

This intuitive framing of the problem of mobile population dynamics has profound implications for research on animal movement, space use, and the niche. The most common approaches to understanding animal space use (collectively, species distribution models, or SDMs; Elith & Leathwick, 2009) approximate an inhomogeneous point process relating animal presence to environmental factors (Johnson, Hooten, & Kuhn, 2013; Renner & Warton, 2013). The coefficients estimated by these models represent relative preference for different resource/habitat types, and the utilization distribution emerging from a SDM is frequently interpreted as the landscape of habitat quality at a given scale (Manly, McDonald, Thomas, McDonald, & Erickson, 2002; Street et al., 2017). As discussed earlier, the implicit assumption to these conclusions is that animals can differentiate between high‐ and low‐quality habitat. However, if animals cannot make such a distinction, then the coefficients and emergent utilization distribution associated with an SDM reflect instead the landscape of perceived quality. The natural and somewhat obvious conclusion is that, at the level of individuals whose presence is likely to be driven at least in part by behavior rather than purely bioclimatic considerations, SDMs measure perceived rather than true habitat quality. Provided there is disagreement between perception and reality, SDMs may be inaccurate, with potentially disastrous consequences for any ecological inference or management decision derived from them.

Our findings suggest that it may be more appropriate and effective to fit models of habitat quality and SDMs immediately following a birthing season when fecundity can be compared to environmental variation so as to overcome any bias introduced by potential perceptive limitations. Yet the question remains, how much does an animal's perceptive capabilities bias an SDM, and how can we accommodate such biases in common statistical methods for SDMs (e.g., MAXENT, logistic regression; Elith & Leathwick, 2009)? We perceive this to be an area of research in need of attention, particularly with regard to conservation of listed species and management of invasive species.

We conclude that there exists a certain scale‐specificity to the limitations of IFD theory. At the scale of patches, we detected oscillations in within‐patch consumer densities corresponding to the timing of reproductive events leading toward the IFD, which we term dynamical facilitation. Following reproduction, the distribution deviated back toward uniformity. This would suggest that, given two landscapes containing different amounts of high‐quality habitat, the landscape with a greater amount of high‐quality habitat will exhibit a higher net density. This should also apply to low mobility scenarios (e.g., Cressman & Křivan, 2006) and competitive exclusion (Kennedy & Gray, 1993; Matsumura et al., 2010), though patch fidelity due to immobility or territoriality would be expected to exacerbate this difference. Thus, despite violations of the assumptions of IFD theory at the scale of animal behavior and decision‐making, an IFD can still arise. At smaller extents (e.g., single landscape or single breeding season), this is driven by local population dynamics within patches producing higher local densities; at broader extents, between‐landscape variation in net quality produces variation in equilibrium densities over time. We already have a term for this phenomenon—the carrying capacity—but this indicates that animal behavior and population dynamics interacting to produce the IFD constitute the mechanism by which carrying capacities emerge (Street et al., 2017). The connection between fine‐scale behavior and equilibrium densities has been demonstrated based on presumed or correlative relationships between behavior and population dynamics (Boyce & McDonald, 1999; Boyce et al., 2016; Matthiopoulos et al., 2015; Street et al., 2015, 2017), but our findings here suggest that the specific interaction between population dynamics and animal movement is the mechanism linking populations across scales and levels of biological organization.

## CONFLICT OF INTEREST

None declared.

## AUTHOR CONTRIBUTIONS

GMS conceived of the project in collaboration with IVE and JTR. IVE wrote the model and prepared figures. GMS led the writing, and IVE and JTR contributed equally to writing and editing.

## DATA ACCESSIBILITY

MATLAB code for the simulations is included in the Online Supplemental Information.
