# Supplementary material for: Dynamical facilitation of the ideal free distribution in nonideal populations
Source: Ecol Evol. 2018 Jan 31;8(5):2471–81. doi: 10.1002/ece3.3811 (PMC5838054; doi:10.1002/ece3.3811)
Supplement: Supplementary file 1 [file ECE3-8-2471-s001.pdf]

Online Supporting Information

Appendix S1: Full results of the 10-patch simulation. In the main text of the paper, Fig. 4 shows the resulting distribution of the individuals at the end of the last dispersal and the last reproductive seasons. Here we include such distributions for all dispersal-reproduction seasons following the initial colonization period and the initial reproductive season (season 1) in Figs. S1–S3.

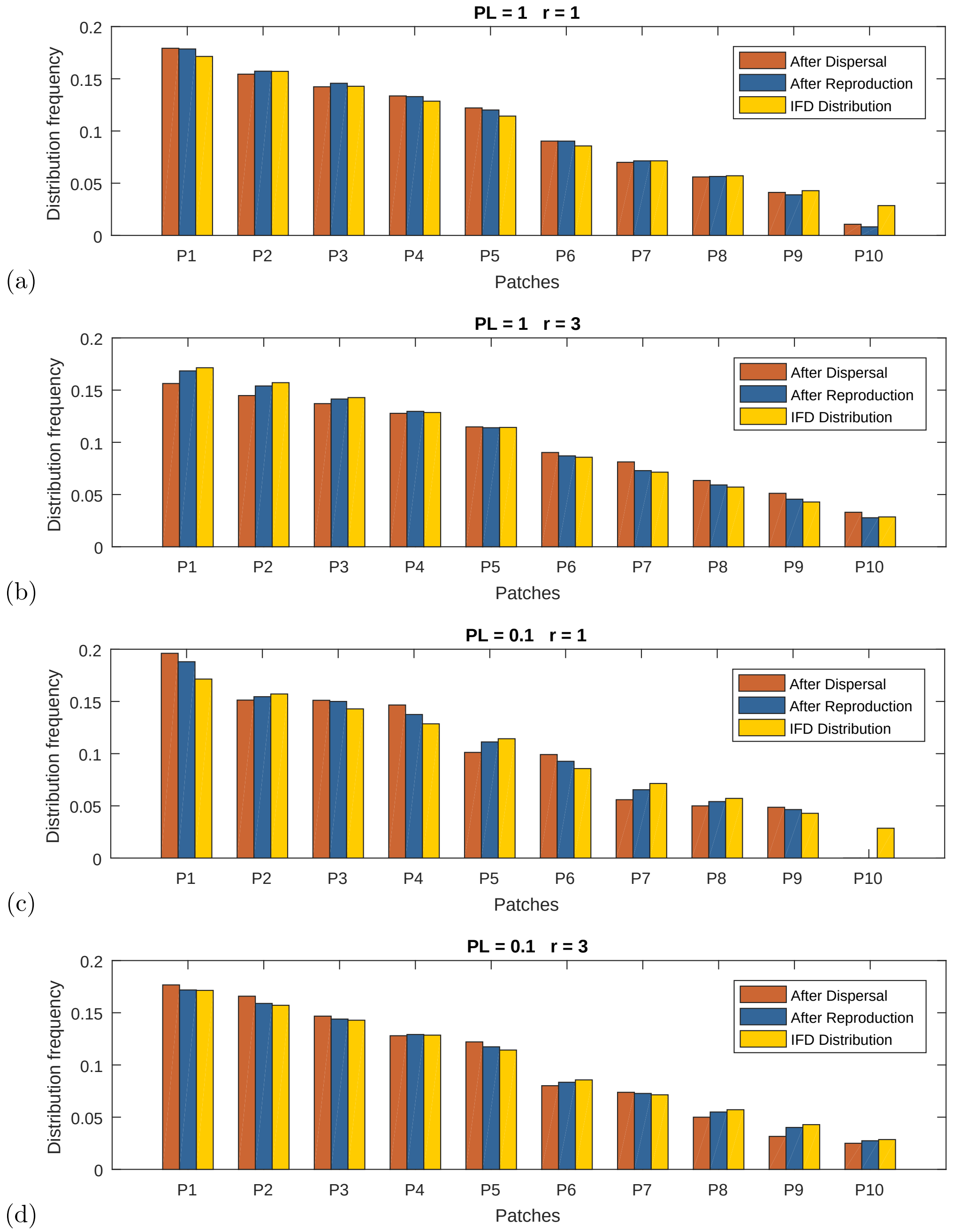

Figure S1. Results of the 10-patch simulation for the exponential growth model at the end of dispersal-reproduction season 2.

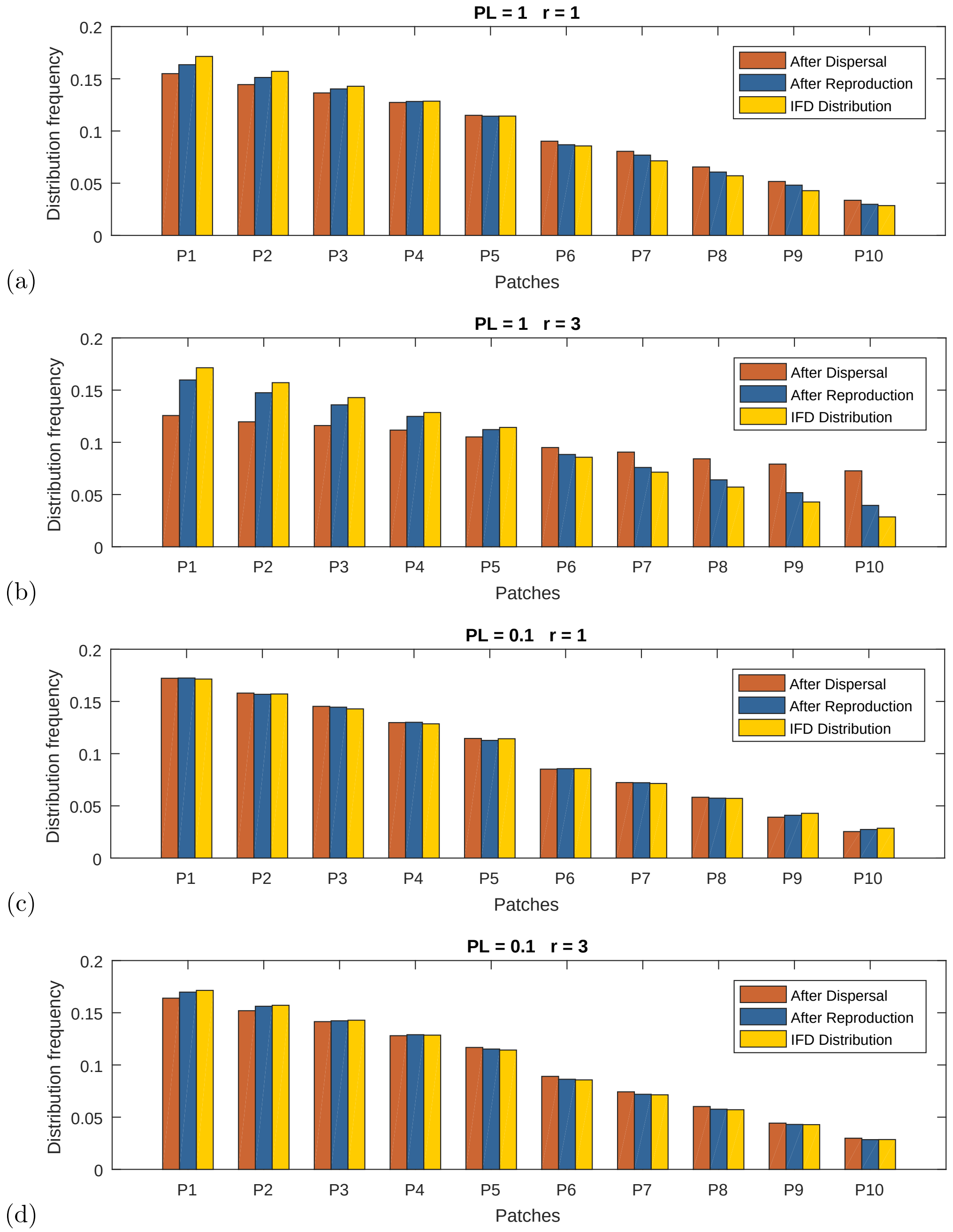

Figure S2. Results of the 10-patch simulation for the exponential growth model at the end of dispersal-reproduction season 3.

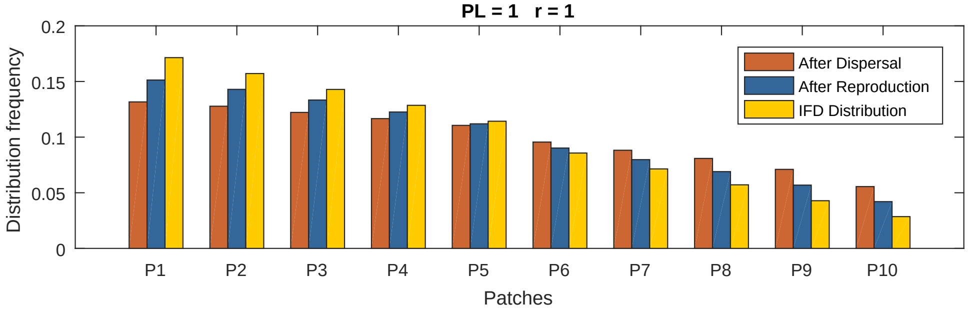

(a)

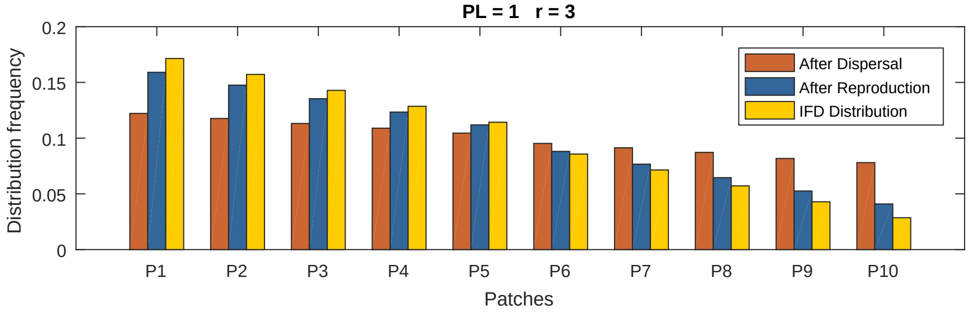

(b)

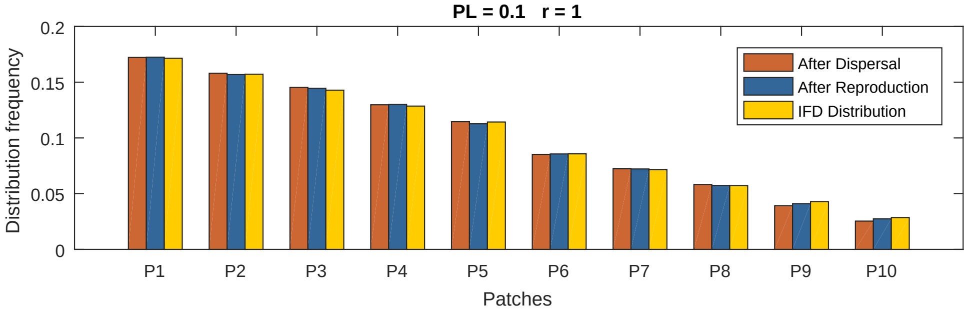

(c)

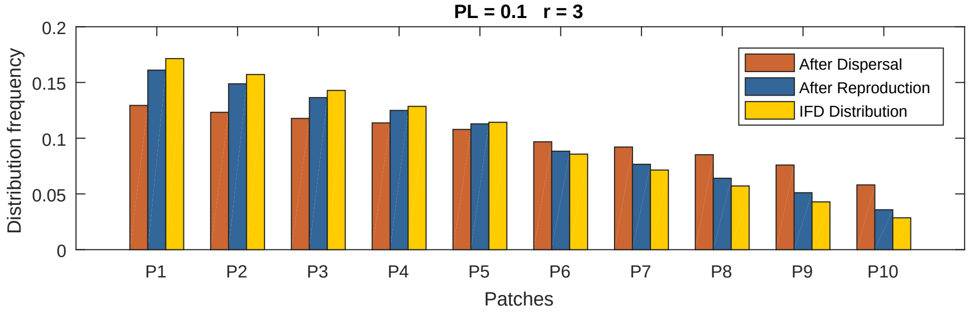

(d)

Figure S3. Results of the 10-patch simulation for the exponential growth model at the end of dispersal-reproduction season 4.

Appendix S2: MATLAB Code. MATLAB code for seasonal exponential growth model.

```
% This is a simulation of discrete patch IFD with population dynamics and
% perception constraints. Population reproduces seasonally at exponential
% rate.

% parameters
npatches = 2; % number of patches
patchres = [12 6]; % allocation of resources between patches
N = 10; % initial size of the population that enters environment
ncensus = 5; % number of reproduction periods
plimit = 1; % perception limit of individuals
r = 3; % reproduction rate defined as the number of reproductive events
% for each individual in the population
totalni = N*(1+r)^ncensus; % total number of individuals at the end
nevents = N*(1+r)/r*((1+r)^ncensus-1); % total number of events
trials = 1000; % number of times simulation will run

% labels
location = 1; % first column of indstat shows patch number of individual
repprop = 2; % second column shows reproduction propensity
cstatus = 1; % first column of snapshot contains the current census number
eventype = 2; % second column of snapshot contains type of the event
init = 0; % label for initialization events
move = 1; % label for movement events
repr = 2; % label for reproduction events

%%%

close all;
pause off;
rng('shuffle'); % reset the random generator seed
tic

for simulations=1:trials

currcensus = 0; % counter for the current peproduction period
currni = 0; % counter showing the number of latest individual
currevent = 0; % counter to track the number of the current event

indstat = zeros(totalni,2); % matrix with individual statistics

patchstat = zeros(1,npatches); % vector holding current allocation of
% individuals between patches

snapshot = zeros(nevents,npatches+2); % this matrix takes snapshots of the
% landspace after every event

payoff = zeros(1,npatches); % potential payoffs of an individual entering
% different patches
```

```
% simulation now begins with Step 1: N individuals enter the landscape one
% at a time following IFD with perception limit

for i=1:N
    currni = currni+1; % new individual is entering the landspace
    currevent = currevent+1; % a new event is going to occur
    payoff = patchres./(patchstat+1); % payoff at potential patches
    maxpayoff = max(payoff);
    goodpatches = abs(payoff-maxpayoff)<=plimit; % logical vector
    % referring to patches that could be chosen by the individual
    indgoodpatches = find(goodpatches==true); % indices of good patches
    if sum(goodpatches)==1
        patchstat(goodpatches) = patchstat(goodpatches)+1; % if only one
        % patch is good, then invidual goes to that patch
        % also update the individual statistics matrix
        indstat(currni,location) = indgoodpatches;
    else
        chosenpatch = randi(sum(goodpatches)); % randomly choose among good
        % patches
        patchstat(indgoodpatches(chosenpatch)) = ...
           patchstat(indgoodpatches(chosenpatch))+1; % put individual into
        % randomly chosen patch among good patches
        % update the individual statistics matrix
        indstat(currni,location) = indgoodpatches(chosenpatch);
    end
    % now take the snapshot of the landspace after every event
    snapshot(currevent,cstatus) = currcensus;
    snapshot(currevent,eventype) = init;
    snapshot(currevent,3:npatches+2) = patchstat;
end

% Step 2 of the simulation is to have the first census in form of rN
% reproduction events. Reproduction propensity is weighed by the actual
% payoff of the individuals

currcensus = currcensus+1; % update the census status
currpopulation = currni; % the number of reproduction events is equal to
% the current population level times reproductive rate
fitness = patchres./patchstat; % compute payoffs at patches
% next, determine the payoff of every individual
for k=1:currpopulation
    indstat(k,repprop) = fitness(indstat(k,location));
end
totalfitness = sum(indstat(1:currpopulation,repprop));

for i=1:r*currpopulation
    currevent = currevent+1;
    rnumber = rand(1); % draw a random number uniformly in (0,1)
    parent = find((cumsum(indstat(1:currpopulation,repprop)) >= ...
        rnumber*totalfitness),1); % find the individual to reproduce
    currni = currni+1; % one more individual enters the scene
```

```
    indstat(currni,location) = indstat(parent,location);
    % puts the newborn into the same location as parent
    patchstat(indstat(parent,location)) = ...
        patchstat(indstat(parent,location))+1;
    % update the statistics of patch distribution
    % and take the snapshot after every event
    snapshot(currevent,cstatus) = currcensus;
    snapshot(currevent,eventtype) = repr;
    snapshot(currevent,3:npatches+2) = patchstat;
end

% now we will perform move/reproduce iterations

for season=2:ncensus

    currpopulation = currni;

    % first, start with movement events
    for i=1:currpopulation
        currevent = currevent+1;
        traveler = randi(currpopulation);
        % randomly choose an individual to move
        ifmovespatchstat = patchstat+1; % create possible scenarios of move
        ifmovespatchstat(indstat(traveler,location)) = ...
            ifmovespatchstat(indstat(traveler,location))-1;
        % no extra individual would appear in the current location
        payoff = patchres./ifmovespatchstat; % payoff at patches if moves
        maxpayoff = max(payoff);
        goodpatches = abs(payoff-maxpayoff)<=plimit; % logical vector
        % referring to patches that could be chosen by the individual
        indgoodpatches = find(goodpatches==true); % indices of good patches
        if sum(goodpatches)==1
            % nothing to do if stays at the current place
            if indgoodpatches~=indstat(traveler,location)
                patchstat(goodpatches) = patchstat(goodpatches)+1;
                patchstat(indstat(traveler,location)) = ...
                    patchstat(indstat(traveler,location))-1;
                indstat(traveler,location) = indgoodpatches;
            end
        else
            chosenpatch = randi(sum(goodpatches));
            % randomly choose among good patches
            % nothing to do if stays at the current place
            if indgoodpatches(chosenpatch)~=indstat(traveler,location)
                patchstat(indgoodpatches(chosenpatch)) = ...
                    patchstat(indgoodpatches(chosenpatch))+1;
                % put individual into randomly chosen patch among
                % good patches
                patchstat(indstat(traveler,location)) = ...
                    patchstat(indstat(traveler,location))-1;
                indstat(traveler,location) = indgoodpatches(chosenpatch);
```

```
            end
        end
        % now take the snapshot of the landspace after every event
        snapshot(currevent,cstatus) = season;
        snapshot(currevent,eventype) = move;
        snapshot(currevent,3:npatches+2) = patchstat;
    end

    % next, do the reproduction phase

    fitness = patchres./patchstat; % compute payoffs at patches
    % next, determine the payoff of every adult individual
    for k=1:currpopulation
        indstat(k,repprop) = fitness(indstat(k,location));
    end
    totalfitness = sum(indstat(1:currpopulation,repprop));

    for i=1:r*currpopulation
        currevent = currevent+1;
        rnumber = rand(1); % draw a random number uniformly in (0,1)
        parent = find((cumsum(indstat(1:currpopulation,repprop)) >= ...
            rnumber*totalfitness),1); % find the individual to reproduce
        currni = currni+1; % one more individual enters the scene
        indstat(currni,location) = indstat(parent,location);
        % puts the newborn into the same location as parent
        patchstat(indstat(parent,location)) = ...
            patchstat(indstat(parent,location))+1;
        % update the statistics of patch distribution
        % and take the snapshot after every event
        snapshot(currevent,cstatus) = season;
        snapshot(currevent,eventype) = repr;
        snapshot(currevent,3:npatches+2) = patchstat;
    end
end

filename = ['ifd_disc_exp_sm_r' num2str(r*100)...
    '_pl' num2str(plimit*100) '_stats.csv'];
dlmwrite(filename, snapshot, '-append');

end

toc
```

MATLAB code for seasonal logistic growth model.

```
% This is a simulation of discrete patch IFD with population dynamics and
% perception constraints. Population reproduces seasonally at logistic
% growth rate.

% parameters
npatches = 2; % number of patches
patchres = [12 6]; % allocation of resources between patches
```

```
N = 10; % initial size of the population that enters environment
plimit = 0.1; % perception limit of individuals
r = 1; % growth rate
K = 100; % carrying capacity
trials = 1000; % number of times simulation will run

% labels
location = 1; % first column of indstat shows patch number of individual
repprop = 2; % second column shows reproduction propensity
cstatus = 1; % first column of snapshot contains the current census number
eventype = 2; % second column of snapshot contains type of the event
init = 0; % label for initialization events
move = 1; % label for movement events
repr = 2; % label for reproduction events

% compute the steps of the logistic growth process and the number of
% associated events
Growth = zeros(1,2); % matrix containing the number of newborns each step
% in the first column (= number of reproduction events), and the total
% number of individuals in the second column (= number of movement events)
% the matrix will grow as we compute the number of reproduction events
cc_thresh = 1/100; % carrying capacity threshold
growth_flag = true; % this will flag to stop reproduction events once
% the population reaches K within cc_thresh
total_pop = N; % counter to track the total population level
i = 1; % counter for the row number of Growth matrix

while total_pop<=K && growth_flag
    newborns = round(r*total_pop*(1-total_pop/K));
    Growth(i,1) = newborns;
    total_pop = total_pop + newborns;
    Growth(i,2) = total_pop;
    i = i+1;
    if abs(total_pop - K)/K <= cc_thresh
        growth_flag = false;
    end
end

% now compute more parameters
[ncensus dummy] = size(Growth); % ncensus = number of reproduction periods
totalni = Growth(ncensus,2); % total number of individuals at the end
nevents = N + sum(Growth(:,1))... % total number of reproduction events
    + sum(Growth(1:(ncensus-1),2)); % total number of movement events


%%%

close all;
pause off;
rng('shuffle'); % reset the random generator seed
tic
```

```
for simulations=1:trials

currcensus = 0; % counter for the current peproduction period
currni = 0; % counter showing the number of latest individual
currevent = 0; % counter to track the number of the current event

indstat = zeros(totalni,2); % matrix with individual statistics

patchstat = zeros(1,npatches); % vector holding current allocation of
% individuals between patches

snapshot = zeros(nevents,npatches+2); % this matrix takes snapshots of the
% landspace after every event
%currevent = 1; % counter for the current event to be used as row number in
% the snapshot matrix

payoff = zeros(1,npatches); % potential payoffs of an individual entering
% different patches

% simulation now begins with Step 1: N individuals enter the landscape one
% at a time following IFD with perception limit

for i=1:N
    currni = currni+1; % new individual is entering the landspace
    currevent = currevent+1; % a new event is going to occur
    payoff = patchres./(patchstat+1); % payoff at potential patches
    maxpayoff = max(payoff);
    goodpatches = abs(payoff-maxpayoff)<=plimit; % logical vector
    % referring to patches that could be chosen by the individual
    indgoodpatches = find(goodpatches==true); % indices of good patches
    if sum(goodpatches)==1
        patchstat(goodpatches) = patchstat(goodpatches)+1; % if only one
        % patch is good, then invidual goes to that patch
        % also update the individual statistics matrix
        indstat(currni,location) = indgoodpatches;
    else
        chosenpatch = randi(sum(goodpatches)); % randomly choose among good
        % patches
        patchstat(indgoodpatches(chosenpatch)) = ...
           patchstat(indgoodpatches(chosenpatch))+1; % put individual into
        % randomly chosen patch among good patches
        % update the individual statistics matrix
        indstat(currni,location) = indgoodpatches(chosenpatch);
    end
    % now take the snapshot of the landspace after every event
    snapshot(currevent,cstatus) = currcensus;
    snapshot(currevent,eventtype) = init;
    snapshot(currevent,3:npatches+2) = patchstat;
end
```

```
% Step 2 of the simulation is to have the first census in the form of
% reproduction events. Reproduction propensity is weighed by the actual
% payoff of the individuals

currcensus = currcensus+1; % update the census status
currpopulation = currni; % the size of the current population
fitness = patchres./patchstat; % compute payoffs at patches
% next, determine the payoff of every individual
for k=1:currpopulation
    indstat(k,repprop) = fitness(indstat(k,location));
end
totalfitness = sum(indstat(1:currpopulation,repprop));

% the number of reproduction events is in the first column of Growth
for i=1:Growth(1,1)
    currevent = currevent+1;
    rnumber = rand(1); % draw a random number uniformly in (0,1)
    parent = find((cumsum(indstat(1:currpopulation,repprop)) >= ...
        rnumber*totalfitness),1); % find the individual to reproduce
    currni = currni+1; % one more individual enters the scene
    indstat(currni,location) = indstat(parent,location);
    % puts the newborn into the same location as parent
    patchstat(indstat(parent,location)) = ...
        patchstat(indstat(parent,location))+1;
    % update the statistics of patch distribution
    % and take the snapshot after every event
    snapshot(currevent,cstatus) = currcensus;
    snapshot(currevent,eventtype) = repr;
    snapshot(currevent,3:npatches+2) = patchstat;
end

% now we will perform move/reproduce iterations

for season=2:ncensus

    currpopulation = currni;

    % first, start with movement events
    for i=1:currpopulation
        currevent = currevent+1;
        traveler = randi(currpopulation);
        % randomly choose an individual to move
        ifmovespatchstat = patchstat+1; % create possible scenarios of move
        ifmovespatchstat(indstat(traveler,location)) = ...
            ifmovespatchstat(indstat(traveler,location))-1;
        % no extra individual would appear in the current location
        payoff = patchres./ifmovespatchstat; % payoff at patches if moves
        maxpayoff = max(payoff);
        goodpatches = abs(payoff-maxpayoff)<=plimit; % logical vector
        % referring to patches that could be chosen by the individual
        indgoodpatches = find(goodpatches==true); % indices of good patches
```

```
        if sum(goodpatches)==1
            % nothing to do if stays at the current place
            if indgoodpatches~=indstat(traveler,location)
                patchstat(goodpatches) = patchstat(goodpatches)+1;
                patchstat(indstat(traveler,location)) = ...
                    patchstat(indstat(traveler,location))-1;
                indstat(traveler,location) = indgoodpatches;
            end
        else
            chosenpatch = randi(sum(goodpatches));
            % randomly choose among good patches
            % nothing to do if stays at the current place
            if indgoodpatches(chosenpatch)~=indstat(traveler,location)
                patchstat(indgoodpatches(chosenpatch)) = ...
                    patchstat(indgoodpatches(chosenpatch))+1;
                % put individual into randomly chosen patch
                % among good patches
                patchstat(indstat(traveler,location)) = ...
                    patchstat(indstat(traveler,location))-1;
                indstat(traveler,location) = indgoodpatches(chosenpatch);
            end
        end
        % now take the snapshot of the landspace after every event
        snapshot(currevent,cstatus) = season;
        snapshot(currevent,eventtype) = move;
        snapshot(currevent,3:npatches+2) = patchstat;
    end

    % next, do the reproduction phase

    fitness = patchres./patchstat; % compute payoffs at patches
    % next, determine the payoff of every adult individual
    for k=1:currpopulation
        indstat(k,repprop) = fitness(indstat(k,location));
    end
    totalfitness = sum(indstat(1:currpopulation,repprop));

    for i=1:Growth(season,1)
        currevent = currevent+1;
        rnumber = rand(1); % draw a random number uniformly in (0,1)
        parent = find((cumsum(indstat(1:currpopulation,repprop)) >= ...
            rnumber*totalfitness),1); % find the individual to reproduce
        currni = currni+1; % one more individual enters the scene
        indstat(currni,location) = indstat(parent,location);
        % puts the newborn into the same location as parent
        patchstat(indstat(parent,location)) = ...
            patchstat(indstat(parent,location))+1;
        % update the statistics of patch distribution
        % and take the snapshot after every event
        snapshot(currevent,cstatus) = season;
        snapshot(currevent,eventtype) = repr;
```

```
        snapshot(currevent,3:npatches+2) = patchstat;
    end
end

filename = ['ifd_disc_logist_K' num2str(K) '_r' num2str(r*100)...
    '_pl' num2str(plimit*100) '_stats.csv'];
dlmwrite(filename, snapshot, '-append');

end

toc
```
